# Hippocampal Atrophy Following Subarachnoid Hemorrhage Correlates with Disruption of Astrocyte Morphology and Capillary Coverage by AQP4

**DOI:** 10.3389/fncel.2018.00019

**Published:** 2018-01-31

**Authors:** Maryam Anzabi, Maryam Ardalan, Nina K. Iversen, Ali H. Rafati, Brian Hansen, Leif Østergaard

**Affiliations:** ^1^Department of Clinical Medicine, Center of Functionally Integrative Neuroscience and MINDLab, Aarhus University, Aarhus, Denmark; ^2^Translational Neuropsychiatry Unit, Department of Clinical Medicine, Aarhus University, Risskov, Denmark; ^3^Sahlgrenska Academy, University of Gothenburg, Gothenburg, Sweden

**Keywords:** astrocyte, aquaporin-4, hippocampus, MRI, subarachnoid hemorrhage

## Abstract

Despite successful management of ruptured intracranial aneurysm following subarachnoid hemorrhage (SAH), delayed cerebral ischemia (DCI) remains the main cause of high mortality and morbidity in patients who survive the initial bleeding. Astrocytes play a key role in neurovascular coupling. Therefore, changes in the neurovascular unit including astrocytes following SAH may contribute to the development of DCI and long-term complications. In this study, we characterized morphological changes in hippocampal astrocytes following experimental SAH, with special emphasis on glia-vascular cross-talk and hippocampal volume changes. Four days after induction of SAH or sham-operation in mice, their hippocampal volumes were determined by magnetic resonance imaging (MRI) and histological/stereological methods. Glial fibrillary acid protein (GFAP) immunostained hippocampal sections were examined by stereological techniques to detect differences in astrocyte morphology, and global spatial sampling method was used to quantify the length density of Aquaporin-4 (AQP4) positive capillaries. Our results indicated that hippocampal volume, as measured both by MRI and by histological approaches, was significantly lower in SAH animals than in the sham-operated group. Accordingly, in this animal model of SAH, hippocampal atrophy existed already at the time of DCI onset in humans. SAH induced retraction of GFAP positive astrocyte processes, accompanied by a significant reduction in the length density of AQP4 positive capillaries as well as narrowing of hippocampal capillaries. Meanwhile, astrocyte volume was higher in SAH mice compared with the sham-operated group. Morphological changes in hippocampal astrocytes seemingly disrupt glia-vascular interactions early after SAH and may contribute to hippocampal atrophy. We speculate that astrocytes and astrocyte-capillary interactions may provide targets for the development of therapies to improve the prognosis of SAH.

## Introduction

Subarachnoid hemorrhage (SAH) accounts for 5% of all strokes and has a high prevalence in relatively young individuals (van Gijn et al., 2007). The overall mortality after SAH is 32–50% (Lin et al., 2014), where 10–15% of patients die before reaching a hospital (Huang and van Gelder, 2002). Meanwhile, the quality of life remains poor for the surviving patients. Permanent neurocognitive dysfunctions such as depression, anxiety, and sleep disorders are common among SAH survivors. Indeed, the majority of those, who survive their SAH, are unable to return to their previous occupation due to long-term impairments of memory, executive and language functions (Broderick et al., 1994). The devastating outcome after SAH is partly ascribed to early brain injury (EBI), which occurs during the first 72 h after SAH and is associated with transient cerebral ischemia, blood–brain-barrier (BBB) disruption, vasogenic edema, and elevated intracranial pressure (ICP) (Friedrich et al., 2012; Fujii et al., 2013). Delayed cerebral ischemia (DCI) is another phenomenon that strongly determines the high mortality and morbidity of patients suffering from SAH. This second phase of brain injury occurs in roughly half of SAH patients between 4 and 14 days after the initial hemorrhage. DCI is associated with deteriorating consciousness and/or focal neurological deficits and can coincide with angiographic evidence of cerebral vasospasm (van Gijn et al., 2007). Despite appropriate management of the aneurysmal hemorrhage and recent advances in reversing vasospasms, the prognosis of SAH has improved little. Therefore, studies to better understand SAH pathogenesis and cellular injury mechanisms behind the degenerative changes that occur after SAH are essential to the development of new treatment strategies and to improve the prognosis of SAH.

Over the past years, astrocytes have attracted considerable attentions in both basic and clinical research, due to their roles in a variety of essential brain functions. Astrocytes are crucial components of neurovascular unit and may hence play a role in the neuronal and vascular dysfunctions following SAH. Astrocyte endfeet surround cerebral vasculature and contribute to normal BBB function (Abbott et al., 2006). Likewise, astrocytic processes reach adjacent synapses, and by this tripartite organization, astrocytes not only sense but also modulate neurotransmission (Harris et al., 2012; Mishra et al., 2016). Reacting to neurotransmitter release, astrocytes are also involved in neurovascular coupling mechanisms that adjust local blood flow to meet metabolic demands (Petzold and Murthy, 2011). In fact, it has recently been shown that astrocytes take part in neurovascular coupling mechanisms seemingly by signaling to the capillary pericytes (Mishra et al., 2016). Astrocyte pathology underlies several neurological and neuropsychiatric disorders (Johnston-Wilson et al., 2000; Sabri et al., 2008), and are thought to be involved in the inversion of neurovascular coupling and edema formation after SAH (Koide et al., 2012; Yan et al., 2012). Sabri et al. (2008) suggested that delayed astrocytic apoptosis might be involved in the development of delayed brain injury after SAH (Sabri et al., 2008).

Aquaporin-4 (AQP4) water channels are highly expressed on the perivascular astrocyte endfeet, indeed, the capillaries network that is covered by astrocytic processes, even not positive for GFAP (Simard et al., 2003) and are also abundant in significant concentration on the astrocyte membranes that ensheath glutamatergic synapses. These bidirectional water channels are thought to be the essential regulators of brain volume (Nagelhus et al., 2004) and play a critical role in rapid and dynamic regulation of extracellular space (ECS) volume (Haj-Yasein et al., 2012; Nagelhus and Ottersen, 2013). Therefore, changes in AQP4 channels can be hypothesized to interfere with neurovascular coupling mechanisms and regulation of brain volume.

Hippocampus is a brain structure, which is crucial for cognitive functions, memory and learning abilities (Shapiro and Eichenbaum, 1999). Accordingly, hippocampal atrophy, as well as cellular alterations at the hippocampus level, can account for some of the SAH-related permanent neurocognitive complications (Kreiter et al., 2002).

In this study, we examined whether astrocyte endfeet and their processes reveal any morphological changes 4 days following experimental SAH and whether signs of neurodegeneration can be detected at this time point. To approach this question, we compared hippocampal volume in fixed, intact brains from a mouse model of SAH and sham-operated animals using a radiological technique (MRI). Then, brains were sectioned and the hippocampal volume was measured histologically. Finally, changes in hippocampal astrocytes and capillary morphology were characterized with a focus on astrocyte processes, in particular, their endfeet that are positive for AQP4.

## Materials and Methods

### Animals

Adult male C57Bl/6 mice (13–15 weeks old, Taconic Bioscience, Inc.) with the mean weight of 27.1 g were used in this study. During all procedures, animals were cared for according to the regulations of the Danish Ministry of Justice and Animal Protection Committees, by permit of the Danish Animal Experiments Inspectorate (Permit no. 2014-15-2934-01004). Experiments were reported according to the ARRIVE criteria (Kilkenny et al., 2010).

### Animal Preparation

All mice were housed in a controlled environment with fixed room temperature (20–22°C) and a 12 h/12 h (light/dark) cycle. Animals had free access to food and water prior to and after the surgery. They were randomly assigned to undergo either SAH induction or a sham surgery by the coin flip. In our study, animals that died during or after the surgery or had secondary ICP raises as a sign of re-bleeding were replaced until the final group size reached (*n* = 7) for both SAH and sham-operated animals. Mice were anesthetized by intraperitoneal injection of 0.5 mg/kg body weight (BW) medetomidine, 5 mg/kg BW midazolam, and 0.05 mg/kg BW fentanyl. Anesthesia was maintained by hourly injections of one-quarter of the initial dose (Thal and Plesnila, 2007). Animals were orally intubated and mechanically ventilated using a SAR-830/AP ventilator (CWE Inc., Ardmore, PA, United States). End-tidal CO_2_ (ETCO_2_) was monitored by a micro-capnograph (Microcapstar, CWE Inc., Ardmore, PA, United States), and core body temperature was maintained at 37°C by using a thermostatically regulated, feedback-controlled heating pad (HB 101/2, Harvard Apparatus, Holliston, MA, United States). Hydration was maintained by intraperitoneal injections of 0.05 ml dextrose 5% (w/v) each hour.

### Intracranial Pressure Measurement

**FIGURE 1:**
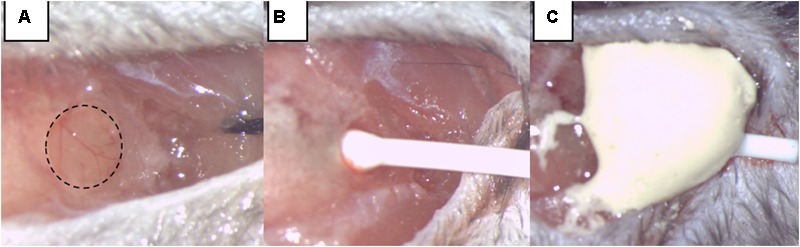
Intracranial pressure (ICP) monitoring. **(A)** A burr hole was made at the junction of the right parietal and temporal bone, extending into the temporal bone by two-thirds of its area. **(B)** The ICP sensor is carefully inserted. **(C)** The ICP sensor was fixed by dental cement.

Intracranial pressure measurement was our mean to monitor SAH induction and any re-bleeding. To insert the ICP sensor (ICP EXPRESS^®^ Monitoring System, DePuy Synthes, Raynham, MA, United States), a hole with a diameter of ∼1 mm was introduced at the junction of the right temporal and parietal bones (**Figure 1**). ICP was then measured in the epidural space of the right hemisphere from approximately 40 min before until 20 min after SAH induction or sham operation. Animals that had secondary ICP increase died (or were euthanized) soon after their re-bleeding and were not included in further investigations.

### SAH Induction by Endovascular Circle of Willis Perforation (CWp)

**FIGURE 2:**
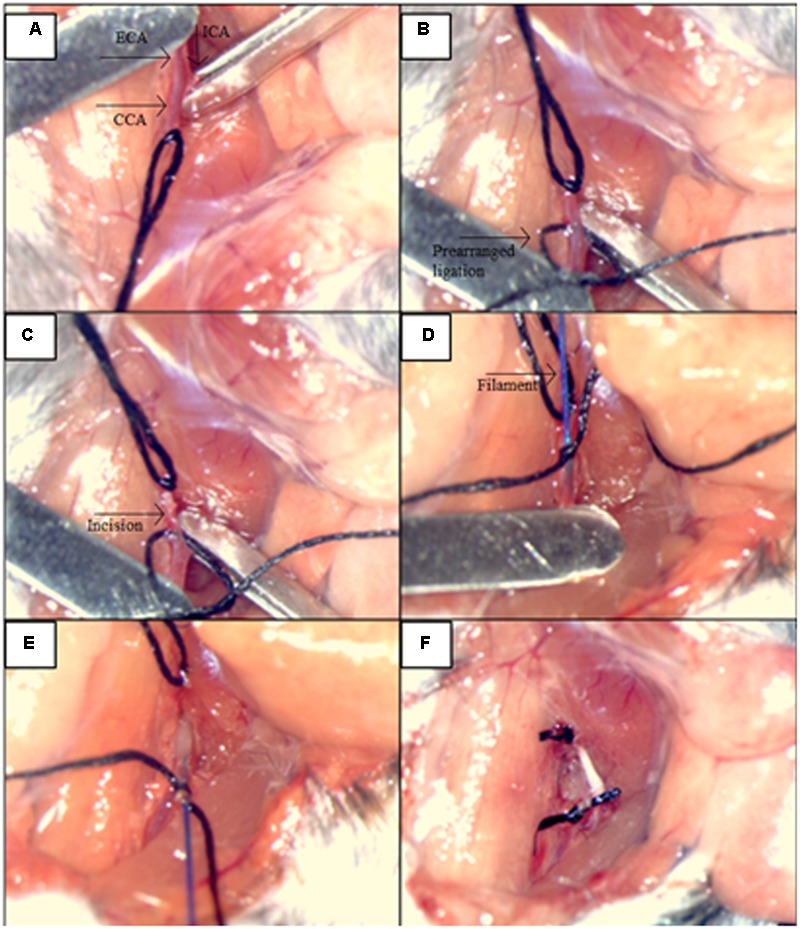
Illustration of the main steps for the procedures: SAH induction by the endovascular circle of Willis perforation (CWp). **(A)** Left external carotid artery (ECA), common carotid artery (CCA) and internal carotid artery (ICA) were exposed. **(B)** ICA and CCA were blocked temporarily with microclips while the ECA was ligated permanently as far cranially as possible. A prearranged ligation was made around the ECA right after the bifurcation of the CCA in order to hold the filament. **(C)** A small incision was cut into ECA for the filament insertion. **(D)** The filament was inserted into ECA, and the insertion part was closed by the prearranged ligation. **(E)** Microclips were removed from CCA and MCA, and the filament advanced toward the skull base via ICA, until a sharp rise in ICP was observed. **(F)** The filament was withdrawn and the ECA was closed by tightening of the prearranged ligation.

Induction of SAH was performed by endovascular perforation of the circle of Willis (CWp model) as previously described (Feiler et al., 2010). Briefly, deeply anesthetized mice were placed in a supine position, and we opened their neck region by a midline incision. After exposing and mobilizing the left common carotid artery (CCA), a 5-0 monofilament was inserted into the left external carotid artery (ECA) and advanced toward the skull base via the left internal carotid artery (ICA). At the branching point of the ICA and the middle cerebral artery (MCA), the filament was advanced further to perforate the vessel, thereby inducing hemorrhage into the subarachnoid space. A sharp rise in ICP indicated successful SAH induction, and the filament was then immediately withdrawn. The immediate retraction of the monofilament right after the ICP rise limited the insertion depth only to perforate the vessel and hence helped to avoid brain injury due to brain tissue being “stabbed” by the filament insertion (**Figure 2**). Sham animals were treated in the same manner, but the filament was not advanced as far as to perforate the vessel and cause hemorrhage. After the surgery and the 20-min monitoring period, anesthesia was terminated by intraperitoneal injection of atipamezole 2.5 mg/kg BW, naloxone 1.2 mg/kg BW, and flumazenil 0.5 mg/kg BW. In order to prevent hypothermia after surgery, animals were kept in a recovery box at 34°C ambient temperature for approximately 24 h. All animals were also subcutaneously injected by standard protocol of three injections of buprenorphine (Temgesic, 0.1 mg/kg/8h) for the pain relief during the first 24 h following the surgery.

### Sample Preparation

Four days following SAH or sham-surgery, deep anesthesia was induced in the animals by an overdose of pentobarbital intraperitoneally. Mice were then transcardially perfused with 4% paraformaldehyde (PFA) (pH = 7.2–7.4). Afterward, brains were isolated by craniotomy and immersed in the same fixative and post-fixed at 4°C prior to MRI. In total, structural MRI data were acquired in 5 SAH brains (two of the total of seven were omitted due to image distortions as the result of blood iron remaining in the tissue after perfusion fixation) and 7 sham brains. Following MRI, the brains were rinsed in PBS and stored in PFA until histological processing. Histological data were obtained from six brains in each group.

### Volumetric MRI Acquisition

For each scan session, a total of five brains were chosen randomly from the two groups so that each scan session contained brains from both groups. Prior to MRI, the brains were washed individually in phosphate buffered saline (PBS) for 24 h to improve the MRI signal by removal of excess fixative. Each brain was then transferred to an MRI compatible tube filled with fluorinert (FC-770, 3M, St. Paul, MN, United States). The samples were placed in a custom-built holder along with a water-filled tube positioned so that brains could be easily identified in the subsequent analysis. The sample holder was then placed in a bore-mounted 40 mm quadrature coil (Bruker biospin, Ettlingen, Germany). High-resolution *T*_2_ weighted anatomical data were acquired using a RARE (rapid acquisition with relaxation enhancement) sequence. Imaging parameters were: echo time (TE) = 12 ms, repetition time (TR) = 8382 ms, in-plane resolution 100 μm × 100 μm, slice thickness 250 μm, and RARE factor = 2. Two averages were acquired, totaling a scan time of 1 h 35 min.

### Tissue Processing

Brains were immersed in cryoprotective (30% sucrose PBS) solution for 48 h, followed by placement on copper blocks for freezing in cold isopentane. Free floating 40-μm coronal sections of brains were collected on a cryostat (Leica, Germany) including tissues between positions bregma -0.94 mm to bregma -4.20 mm, containing the hippocampus. Of note, performing our histological volumetry investigations on these cryostat sections had the advantages of the negligible amount of shrinkage in the x- and y-axis. Three series of sections were chosen based on a systematic sampling principle and a section-sampling fraction of 1/8 (Gundersen, 2002) by selecting the first section of each series randomly. One set of sections was hematoxylin and eosin (H&E) stained in order to quantify capillaries and the hippocampus volume with light microscopy. The rest of the series were labeled using primary antibodies for three-dimensional (3D) reconstruction of astrocytes.

### Immunohistochemistry

Free-floating sections were washed three times in Tris buffer saline (TBS) containing 0.1% Triton X-100 (TBS-T). Each wash lasted 5 min. Afterward, sections were rinsed in boiled antigen retrieval solution (Dako, Ref# S1699, Denmark) dissolved in distilled water in a microwave oven for 30 min. The sections were then allowed to cool down to room temperature (30 min) in the same solution. Sections were washed 2 min × 5 min with TBS-T and 1 min × 5 min with TBS. In the next step, blocking endogenous peroxidase (30% H_2_O_2_ and methanol dissolved in TBS) was performed for 15 min. Thereafter, sections were washed 1 × 10 min with TBS and 1 × 10 min with TBS-T. Following this, one series of sections was incubated with a polyclonal rabbit anti-GFAP (Dako, Ref# Z0334, Denmark) at 1:500 dilution and another series was incubated with a polyclonal rabbit anti-Aquaporin 4 (Boster Biological Technology, Ref#PB9475). All primary antibodies were diluted in 0.05 M TBS, 1% Triton X-100 and 0.2% non-fat dry milk and incubated overnight at 4°C. Subsequently, sections were washed in TBS 3 min × 10 min and then incubated in polyclonal secondary goat anti-rabbit IgG antibody/HRP (1:200 dilution; Dako, Ref# P0448, Denmark) for 2 h. Sections were then rinsed in TBS-T 3 × 10 min. Immunolabelling was performed using 3,3′-diaminobenzidine (DAB) solution (Sigma, United States) for 2 min followed by rinsing in distilled water for 3 min × 1 min. Finally, sections were mounted on gelatin-coated slides and GFAP stained sections were stained with 0.25% thionin solution (thionin, Sigma T3387).

### Hippocampal Volume Measurement on Histological Sections

**FIGURE 3:**
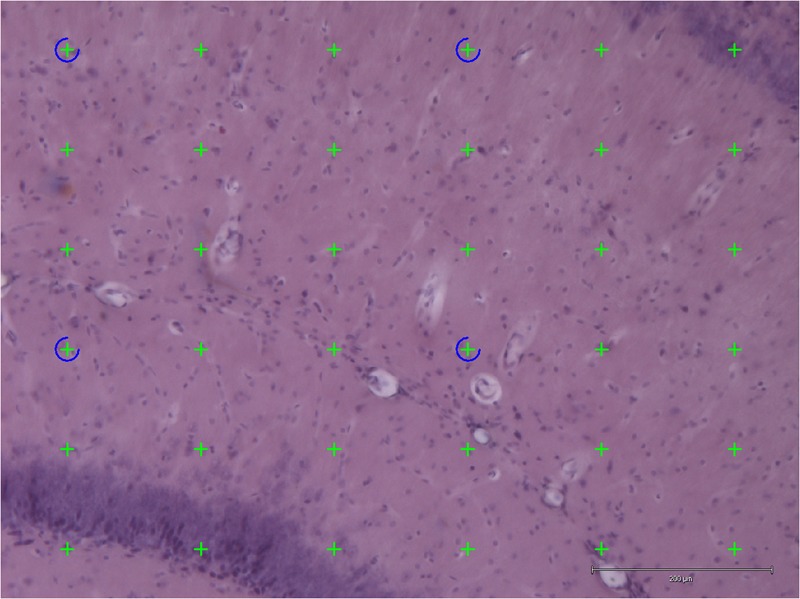
Application of point counting on 40-μm thick coronal H&E-stained sections of the hippocampus for measuring the volume of an area. Thirty-six green points hit the area and were counted. Scale bar = 200 μm.

Stereology was performed using an Olympus light microscope (Olympus BX53, Olympus, Denmark) modified for stereology with a digital camera (Olympus DP73, Olympus, Denmark), a motorized microscope stage (Prior H101 with controller PS3J100, Cambridgeshire, United Kingdom), and a 10× lens (Olympus, Splan, N.A. 0.45). Using this setup, the hippocampal volume was estimated from the H&E stained sections by applying the Cavalieri estimator with point counting (**Figure 3**) (Gundersen et al., 1988) using the newCAST software (Visiopharm Integrator System, version 5.0.1.112, Visiopharm, Hørsholm, Denmark).

The points were superimposed on the sections of hippocampus with the equal space and the number of points hit the area of interest (hippocampus) were counted.

The following formula was applied for estimating the volume of the hippocampus:

$$\text{V=}\sum P\cdot (\frac{a}{p})\cdot T\cdot \frac{\text{1}}{\text{SSF}}$$

where *ΣP* is the total number of the points hitting the hippocampus; *(a/p)* is the area per point; *T* is the section thickness (40 μm); *SSF* is the section sampling fraction.

### MRI-Based Hippocampal Volumetry

**FIGURE 4:**
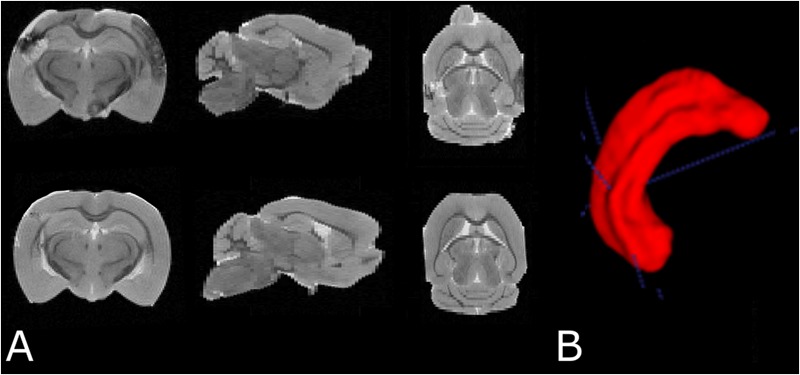
**(A)** Examples of *T*_2_ weighted MRI data demonstrating slices in three orthogonal planes from the fixed brain of an SAH animal (top row) and a sham-operated animal (bottom row). **(B)** 3D segmentation of the left hippocampus. The cortical abnormality seen in the SAH brain is due to the contralateral pressure probe used to monitor ICP.

Data were exported to ITK snap (Yushkevich et al., 2006) for manual hippocampus segmentation. Hippocampus volume was estimated in each brain by voxel counting followed by conversion to physical volume (mm^3^) using the 3D MRI resolution (**Figure 4**).

### Quantification of Hippocampal Capillary Diameter

The capillary diameter was estimated in the molecular layer of the dentate gyrus (MDG), a portion of the hippocampus, which is densely vascularized. The delineation of the MDG was performed on a light microscope by using a 4× objective (Olympus, UPlanSApo, N.A. 0.16) at a magnification of 183×. For analyses of hippocampal vascular diameter, 80–100 capillaries per animal were sampled randomly using an optical disector probe with a height of 20 μm using the newCAST software (Visiopharm, Hørsholm, Denmark). The capillary diameter was determined with a 100× oil-immersion objective lens (Olympus, Plan Apochromat, N.A. 1.25) and the measurement was only performed when the outer wall of the capillary was in focus and fell entirely or partially inside the unbiased counting frame without crossing the exclusion lines of the frame.

### Quantification of the Length Density of AQP4 Positive Capillaries in the Hippocampus

The length density of AQP4 positive capillaries in the hippocampus was estimated using the global spatial sampling method (Larsen et al., 1998) with a 60× oil immersion lens (Olympus, Plan Apochromat, N.A. 1.35) on the immunostained sections for AQP4. In a 3D sampling box, isotropic virtual planes with a fixed plane separation distance (*d* = 25 μm) were systematically and randomly superimposed on the area of interest. The total number of intersections between the virtual planes and the AQP4 positive capillaries was used to estimate the length density of the AQP4 positive capillaries. The height of the counting box was 20 μm with a top guard zone of 5 μm. Based on the size of the counting frame area and the box height, about 200 capillary intersections per animal were counted.

The following formula was applied for measuring the length density of the AQP4 positive capillaries:

$$L_{\text{V}}=\frac{2\cdot p(box)}{avga(plane)}\cdot \frac{\sum Q}{\sum P}$$

where *L*_v_ is the length density of the AQP4 positive capillaries; *ΣQ* is the sum of intersections between the test lines and the capillaries; *p(box)* is the number of box corners (4); avg a (plane) is the average of the plane area; *ΣP* is the sum of the box corners hitting the area of interest.

### Image Acquisition of Astrocytes in the Hippocampus

A systematic set of Z-stacks of the 40-μm thick GFAP stained sections of the hippocampus were captured by an Olympus scanner VS120 (using software VS-ASW 2.5, Build 9483) by using a 60× oil objective (Olympus, Splan, N.A. 1.35). The height of the Z-stacks was 20 μm and images were acquired in steps of 0.5 μm (**Figure 7**).

### 3D Reconstruction of the Astrocytes in the Hippocampus

The morphology of the hippocampal astrocytes was quantified in terms of the number of branches, total branch length, and the Sholl intersections. This was done using the Filament Tracers algorithm of the Imaris software (Version 7.7, Bitplane A.G., Zurich, Switzerland) on the images captured with an Olympus scanner (Olympus VS120, Olympus Inc., Tokyo, Japan). The complexity of astrocytic processes was quantified using Sholl analysis (Sholl, 1953), by selecting the branch endpoints and bifurcations. This type of analysis is done in three dimensions. The total length of astrocyte processes was measured radially in 10-μm steps using the center of the astrocyte soma as a reference point. The sum of the number of intersecting astrocytic processes for all circles was quantified for each astrocyte, and the mean of the results from 20 astrocytes per animal was calculated. Moreover, the volume of the GFAP positive astrocytes in the hippocampus was measured by applying the 3D nucleator (Gundersen, 1988) with a 100× oil-immersion objective lens. The mode was vertical uniform random (VUR) based on the assumption of rotational symmetry of the astrocytes. Specifically, we assumed rotational symmetry around the vertical axis perpendicular to the hippocampus subregion. In order to calculate unbiased astrocyte volume with VUR nucleator, sampling must be performed on VUR sections (Gundersen, 1988). However, due to the complexity of hippocampal subregions structure and the diffuse damage in brain regions, including the hippocampus in this animal model, we were limited to perform measurements on the coronal sections of hippocampus based on the assumption of rotational symmetry around the vertical axis perpendicular to the area of interest. Consequently, the cell volume measurements may contain a minor bias. However, since identical cutting planes and sampling method were used for all brains in both groups of animals, this bias would be expected to be similar in the two groups. Six half-lines were selected, and approximately 50-80 astrocytes were randomly sampled in each animal using the optical disector with a height of 15 μm. For reliable reconstruction of astrocyte morphology, the selected astrocytes must fulfill three criteria: (1) the astrocyte soma must be located in the middle of the section thickness with a clear border; (2) the astrocytic process must be intact; and (3) the branches of the selected cell should be readily visible, without any tangling with branches of nearby cells or haziness from the background staining.

### Statistical Analysis

All data were analyzed using SPSS (IBM Corp. Released 2013, Version 22.0. Armonk, NY, United States). Graphs were created using Sigmaplot 12.5 (SYSTAT, San Jose, CA, United States). Prior to statistical tests, normal distribution of data was ensured by making a Q–Q plot of the data. The variance homogeneity of data was also examined by Levene’s test. If the distribution of data was not normal and/or data variance was different, a logarithmic transformation was employed before statistical testing. The continuous morphological data from the two groups (SAH and sham) was compared using the independent *t*-test. The total number of astrocytic branches was compared using the Mann–Whitney test. Two-tailed Pearson analysis was used to test the correlation between different morphological parameters of the hippocampus. In all cases, the significance level was set at *p* < 0.05. The results are presented as mean ± standard deviation.

## Results

### ICP Monitoring

**FIGURE 5:**
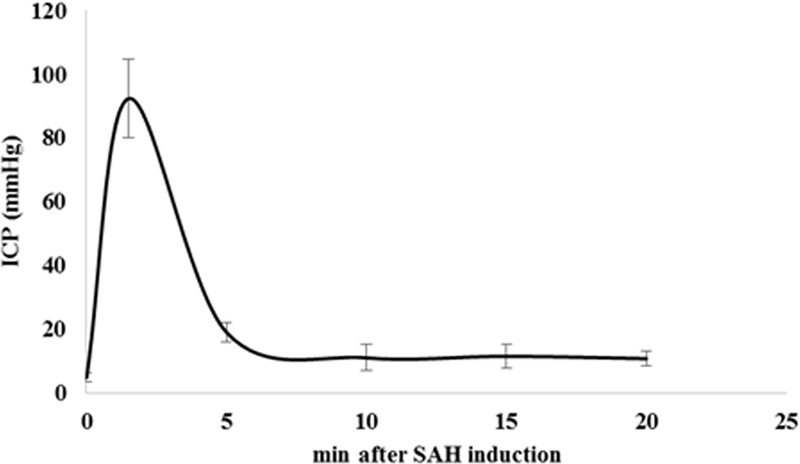
Time course of ICP changes after SAH induction. SAH induction was proven and controlled by continuous monitoring of ICP. We observed a prompt and sharp ICP increase in SAH animals up to 89 ± 17 mmHg indicating SAH induction. ICP values dropped to 11.5 ± 2 mmHg after 5 min of SAH induction and remained almost stable during next 15 min of monitoring. Values are given in mean and standard deviation. SAH, subarachnoid hemorrhage; ICP, intracranial pressure. Adopted from [Anzabi et al., in second review, microcirculation research (2018)].

Intracranial pressure increased promptly and reached to 89 ± 17 mmHg in response to the SAH induction. Within 5 min after the hemorrhage, ICP values dropped to 11.5 ± 2 mmHg and stayed almost constant within the next 15 min of monitoring (**Figure 5**).

### Capillary Diameter in the Hippocampus

**FIGURE 6:**
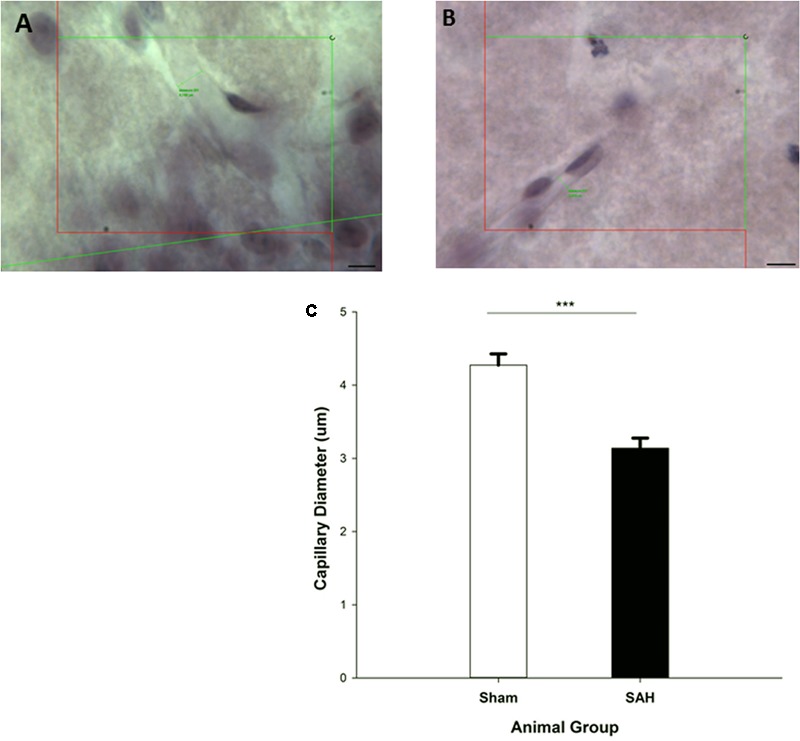
Diameter measurement of H&E stained capillaries within the Molecular-layer of dentate gyrus of the hippocampus. The small green lines in **(A,B)** are examples of capillary diameter measurement with no cell soma inside of the counting frame. **(A)** Sham mouse and **(B)** SAH mouse. **(C)** The diameter of hippocampal capillaries between SAH and sham groups. SAH: subarachnoid hemorrhage; ^∗∗∗^*p* < 0.001. Scale bar = 10 μm.

We observed a significant difference in the capillary diameter between the sham and SAH groups (*p* < 0.001), with SAH mice exhibiting a significant narrower mean capillary diameter compared with the sham group (3.13 ± 0.13 μm vs. 4.27 ± 0.15 μm, respectively) (**Figure 6**).

**FIGURE 7:**
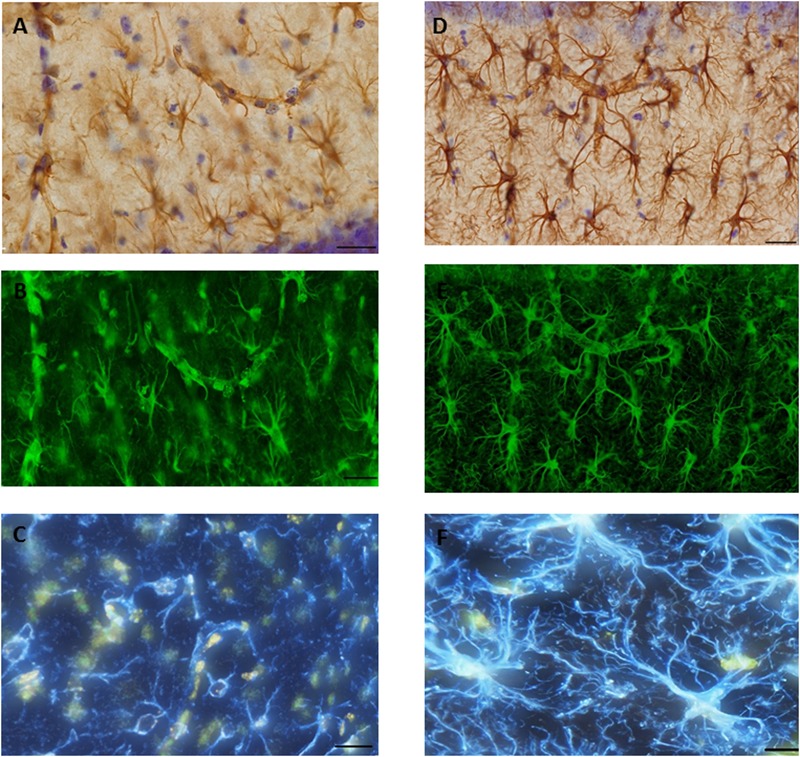
Glial fibrillary acid protein (GFAP) immunostaining image was captured from the hippocampus of **(A)**. SAH and **(D)** sham mice hippocampus using a 60× objective with ultimate resolution from light microscopy. GFAP positive astrocytes were tracked and analyzed based on the processes terminal points using Filament Tracers Algorithm in Imaris software. Tissue includes different parameters (cell soma and branches). Each channel (color) is related to one parameter, therefore selecting one channel resulted into the same (green) color for both branches and cell soma in **(B)**. SAH and **(E)** sham, two channels (blue is related to the branches; yellow is related to the soma) in **(C)**. SAH and **(F)** sham. Scale bar = 20 μm.

### Hippocampal Volume

**FIGURE 8:**
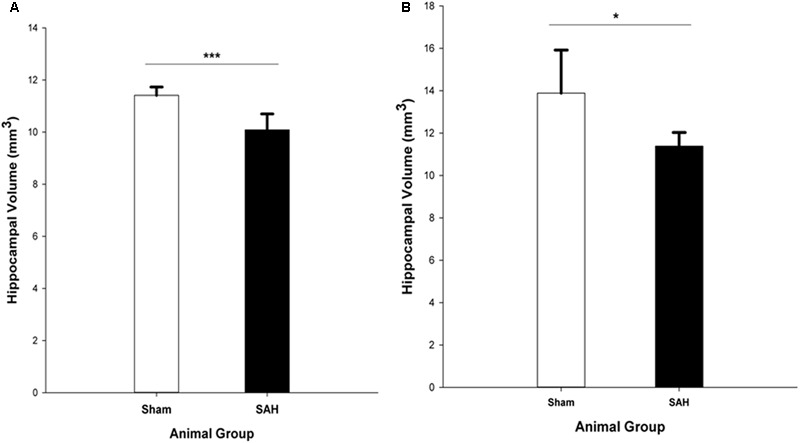
Total volume of the hippocampus in SAH and sham groups 4 days following surgery, obtained from **(A)**. H&E stained sections (Cavalieri estimator method) and **(B)**. MR images segmentation. SAH, subarachnoid hemorrhage; ^∗^*p* < 0.05, ^∗∗∗^*p* < 0.001.

The stereological assessment of the hippocampal volume (Cavalieri estimator) showed a significant difference between SAH and sham groups (*p* < 0.001), with a remarkable decrease in mean volume of the hippocampus in the SAH group compared with sham animals (10.08 ± 0.60 mm^3^ vs. 11.41 ± 0.31 mm^3^, respectively) (**Figure 8A**). Our findings showed a significant positive correlation between the reduction in the diameter of capillaries and the smaller size of the hippocampus (*r* = 0.82, *p* < 0.001).

The MRI analysis of ipsilateral hippocampus volume in the intact brains revealed significantly lower hippocampal volume in the SAH group vs. sham group (*p* = 0.017) (**Figure 8B**).

### Morphological Analysis of Hippocampal Astrocytes

#### Total Length of Astrocytic Processes

**FIGURE 9:**
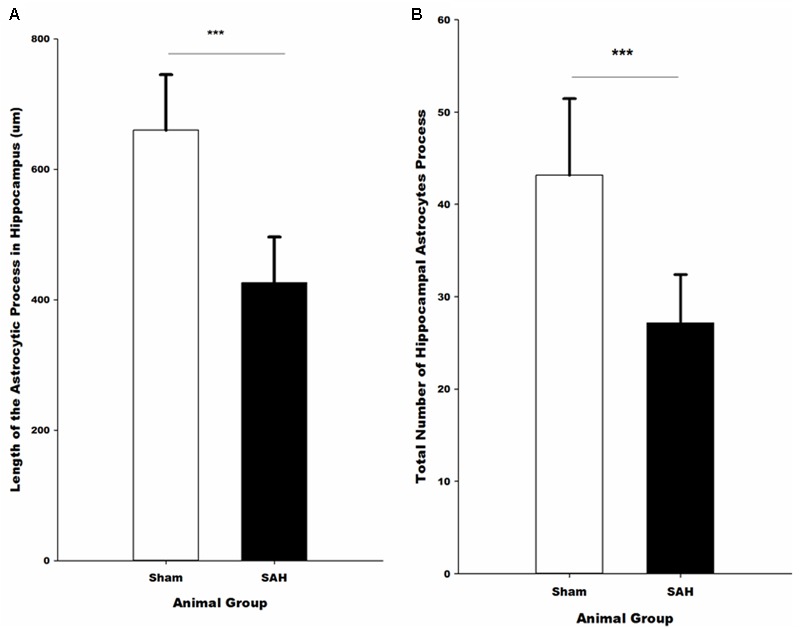
Quantitative analysis of hippocampal astrocytes. **(A)** The length of the astrocytic processes and **(B)** the number of astrocytic processes in SAH and sham mice 4 days following the surgery. SAH, subarachnoid hemorrhage; ^∗∗∗^*p* < 0.001.

Morphological analysis of hippocampal GFAP positive astrocytes showed significant changes in the length of the astrocytes branches 4 days after SAH induction with significantly decreased astrocytic branch length (*p* < 0.001) in the SAH group compared with the sham group (426 ± 70 μm vs. 660 ± 85 μm) (**Figure 9A**). Furthermore, our results demonstrated a positive correlation between the decreased total length of the astrocytic processes and the volume of the hippocampus and diameter of capillaries, respectively (*r* = 0.751, *p* = 0.005; *r* = 0.772, *p* = 0.003).

#### Total Number of Astrocytic Processes

The total number of the hippocampal astrocytic branches was significantly influenced by SAH induction and showed a significantly lower number of branches in the SAH mice in comparison with the sham group (*p* < 0.001) (**Figure 9B**). Correlation analysis demonstrated a significant association between the total number of astrocytic branches and the total length of astrocytic processes (*r* = 0.957, *p* = 0.000). Furthermore, correlation analysis showed that the decreased volume of hippocampus and diameter of capillaries had a positive correlation with the total number of astrocytic branches (*r* = 0.731, *p* = 0.007; *r* = 0.690, *p* = 0.013).

#### Sholl Analysis

**FIGURE 10:**
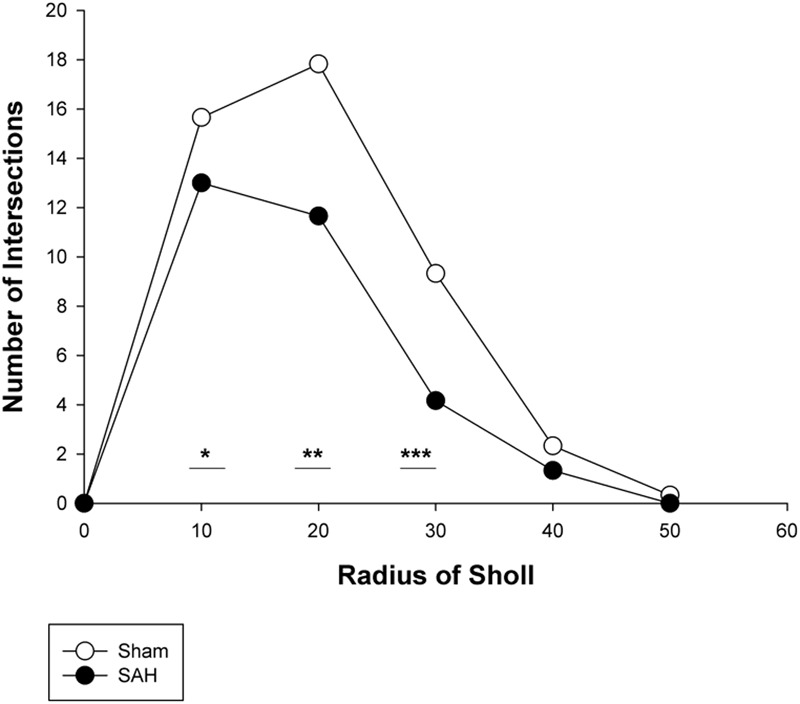
Branching pattern alterations of hippocampal astrocytes using Sholl analysis in the SAH and sham groups. The number of branching intersections at 10, 20, and 30 μm away from the cell soma was significantly lower in the SAH group vs. sham group. SAH, subarachnoid hemorrhage; ^∗^*p* < 0.05, ^∗∗^*p* < 0.01, ^∗∗∗^*p* < 0.001.

To further evaluate the morphological alteration of astrocytes 4 days following SAH, we compared the branching patterns of astrocytes in the hippocampus. The results showed a significant reduction in the astrocytic arborization 4 days following SAH induction compared with the sham group with the significantly lower number of branching intersections from the cell soma at distances of 10 μm (*p* = 0.03), 20 μm (*p* = 0.002), and 30 μm (*p* = 0.000). No difference was observed between the SAH and the sham groups within 40 and 50 μm Sholl spheres (*p* > 0.05) (**Figure 10**).

#### Volume of Astrocytes

**FIGURE 11:**
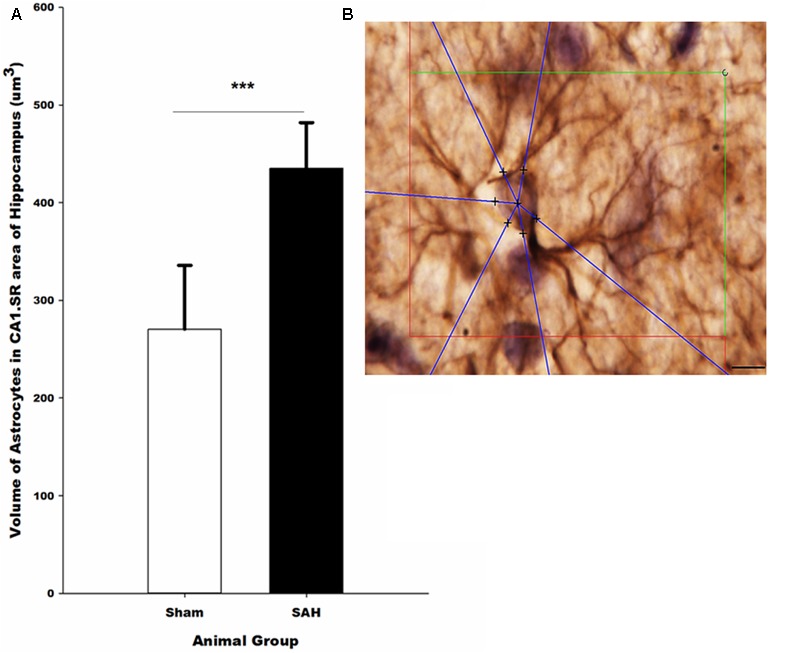
**(A)** Volume of the astrocytes in the hippocampus in SAH and sham groups 4 days following the surgery on GFAP immunostained sections. **(B)** Illustration of the 3D nucleator tool for the measurment of the volume of GFAP positive astrocytes in the hippocampus. The intersections between six half-lines (blue half-lines) and the border of the cell soma were specified by using a 100× objective. Scale bar is 20 μm; ^∗∗∗^*p* = 0.001. Scale bar = 10 μm.

The 3D analysis of astrocyte size showed that the volume of the GFAP positive astrocytes in the hippocampus was significantly altered 4 days after SAH induction. Specifically, significantly larger astrocytes were observed in the SAH group (435 ± 47 μm^3^) compared with sham group (270 ± 66 μm^3^, *p* = 0.001) (**Figure 11**). Notably, our results showed that at day 4 after SAH, the size of the hippocampal astrocytes was negatively correlated with the other parameters that describe astrocyte morphology including the length and number of astrocyte branches (*r* = -0.624, *p* = 0.03; *r* = -0.586, *p* = 0.045). Additionally, we found a significant negative correlation between the increase in the volume of the astrocytes and reduction in the hippocampal volume and capillary diameter decrease (*r* = -0.665, *p* = 0.01; *r* = -0.876, *p* = 0.000). The negative correlation was stronger between the volume of astrocytes and capillary diameter compared with other hippocampal morphological parameters.

### Length Density of AQP4 Positive Capillaries in the Hippocampus

**FIGURE 12:**
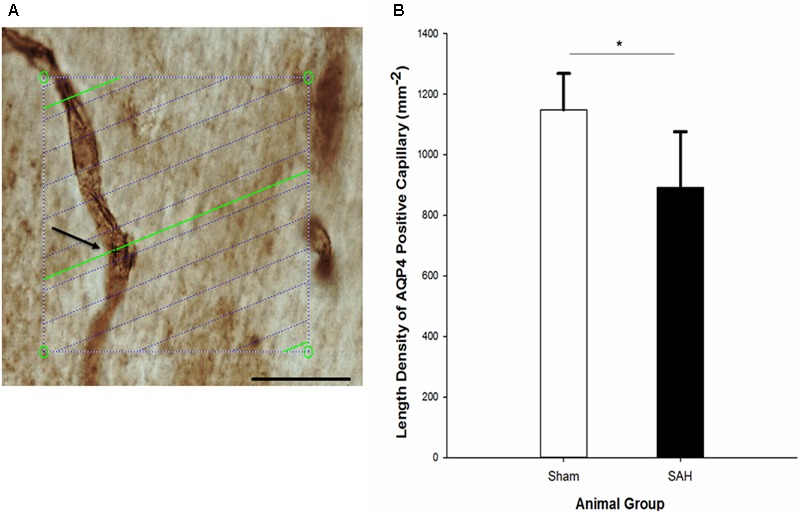
**(A)** Estimation of the length density of AQP4 positive capillaries in 40 μm thick AQP4 immunostained section of hippocampus on the light microscope connected to newCAST software with a 60× oil immersion lens. The green lines represent the intersection between isotropic virtual plane and the focal plane. Whenever the AQP4 positive capillaries are in focus and the virtual planes intersect them, they are counted. The intersection between a green line and one AQP4 positive capillary is shown by black cross. The four box corner points are used to estimate the reference volume. Scale bar, 30 μm. **(B)** Length density of the AQP4 positive capillaries in the hippocampus of the SAH and the sham groups 4 days following the operation. SAH, subarachnoid hemorrhage; ^∗^*p* < 0.05. Scale bar = 20 μm.

Assessment of hippocampal AQP4 positive capillaries showed a significant difference in the length density of AQP4 positive capillaries between the SAH and sham groups. Substantial reduction in the length density of AQP4 positive capillaries 4 days following SAH induction was found (*p* = 0.01) (**Figure 12**). Pearson analysis indicated a significant positive correlation between the reduction in length density of AQP4 positive capillaries and decrease in both capillary diameter and the length of astrocytic processes (*r* = 0.702, *p* = 0.01; *r* = 0.594, *p* = 0.04). However, neither the correlation between length density of AQP4 positive capillaries and astrocyte volume nor AQP4 positive length density and volume of the hippocampus were significant (*p* > 0.05).

## Discussion

The main findings of our study are (i) histological evidence of changes in astrocytic morphology and (ii) neuroimaging as well as stereological evidence of hippocampal atrophy 4 days after SAH induction in a realistic animal model, in the transitional time between early and delayed brain injury in humans. Specifically, hippocampal astrocytes swelled in the cell bodies, retracting their processes and reducing the capillary coverage by AQP4 positive astrocyte endfeet. Both hippocampal capillary diameter and the length density of AQP4 positive capillaries were reduced.

In the current study, GFAP and AQP4 immunostaining were used to label astrocytic processes in the hippocampus. Of these, GFAP was used to investigate the alteration of astrocytic processes as well as cell bodies, and hence to address SAH-related astrocyte structural changes affecting astrocytes’ participation in tripartite synapses. Meanwhile, astrocytic interactions with capillaries were investigated using AQP4 labeling (Nielsen et al., 1997) due to the high expression of AQP4 in the endfeet of astrocytes surrounding the blood–brain barrier (BBB) (Amiry-Moghaddam and Ottersen, 2003). Indeed, the proper function of BBB requires 90% coverage of brain capillaries by the astrocytes endfeet (Jukkola and Gu, 2015).

Accordingly, we observed reduced coverage of capillaries by AQP4 positive astrocyte endfeet in the hippocampus 4 days after SAH induction compared with the sham group. A reduction in the coverage of hippocampal capillaries by AQP4 positive astrocyte endfeet can either be the result of displacement of AQP4 from the endfeet, or be due to retraction of astrocyte endfeet from their normal pericapillary positions, or be ascribed to lower expression of the AQP4 protein. Previously, it has been shown that the mislocalization and not lower expression of the AQP4 protein is correlated with the delayed onset of imminent brain edema (Vajda et al., 2002). We did not assess the total AQP4 protein levels in our study and hence cannot rule out the possibility that lower abundance of brain AQP4 protein might explain our findings. However, we observed retraction of astrocytic processes accompanying with hippocampus atrophy and not edema 4 days after SAH. Accordingly, we believe that the reduction of the AQP4 positive capillaries is more likely a result of retraction of astrocyte endfeet from their normal pericapillary positions. Indeed, it has been indicated that astrocytes experience both morphological and functional plasticity in response to the ischemic insult. The neurovascular unit also undergoes intracellular and extracellular edemas, where BBB disruption adds to the severity of the disturbance in the homeostasis of the neurovascular unit (Wang and Parpura, 2016). Consistent with the results of the present study, it has been shown that astrocytes experience glial retraction as ischemia progresses. This reaction can hence disrupt the integrity of the BBB directly and could be expected to deteriorate the ischemic stress after SAH (Frydenlund et al., 2006; Steiner et al., 2012). In parallel with the mentioned morphological plasticity, astrocytes also experience functional plasticity by changing the expression of ion and water transport proteins including GFAP and AQP4 (Wang and Hatton, 2009). These components are indeed believed to be the key determinant of the structural and functional plasticity, in particular, via edema formation. Accordingly, the integrity of the BBB and homeostasis of the neurovascular unit depend critically on the morphological and functional plasticity of astrocytes also in connection with GFAP and AQP4 (Wang and Parpura, 2016).

Of note, the thickness of cortical astrocyte endfeet was examined by McConnell et al. (2016) 48 h following SAH in mice. They concluded that capillary flow disturbance is not the consequence of astrocyte endfeet swelling, nor constriction of pericytes (McConnell et al., 2016). Our findings indicate that, at least in the hippocampus, loss of capillary coverage by AQP4 positive astrocytes and retraction of astrocyte endfeet, may affect capillary flows by disrupting neuro-capillary coupling mechanisms (see below) and BBB disruption.

We found an increase in the volume of hippocampal astrocytes 4 days after SAH. It has been demonstrated that, astrocyte swelling mainly depends on the osmotic gradients caused by abnormal transport of ion and water across the cell membranes (Larsen et al., 2014).

In light of AQP4 key role in the regulation of brain water flux (Nagelhus and Ottersen, 2013), we can expect that loss of capillary coverage by AQP4 positive astrocyte endfeet, may interfere with water homeostasis across the astrocyte membrane. Swelling of the astrocytic soma in SAH mice hence could be related to the dysregulation of water transport across their cell membranes (Barres et al., 1990; Walz, 1992; Bender et al., 1998) resulting in the altered osmotic gradients (Larsen et al., 2014).

Hippocampal capillary diameter in SAH group was reduced compared with the sham animals. We further observed that the reduced diameter of capillaries was positively correlated with the morphological alteration of astrocytes. Therefore, we had a reason to speculate that functional asthenia of astrocytes, as a result of the observed structural changes, can lead to substantial decrease in the diameter of hippocampal capillaries, and consequently dysregulation of microcirculation in SAH mice.

Neuropsychological and neurocognitive complications, including depression, memory deficits, and learning difficulties, are commonly ascribed to the secondary brain injury after SAH (Thompson et al., 2011). In this study, we found evidence of pathological changes that might cause SAH related neurocognitive and neuropsychological deficits. Accordingly, profound cellular alterations were observed in the hippocampus following SAH and both neuroimaging and stereological measurements of the hippocampal volume revealed a significant reduction in the hippocampal volume 4 days after SAH induction in comparison with the sham group. Our neuroimaging findings were also consistent with the former MRI studies in SAH patients, where they demonstrated the reduction in the brain volume and especially smaller size of hippocampus 1 year after SAH onset (Bendel et al., 2006, 2010). Taken together, these findings offer support for the use of MRI-based hippocampal volume investigations after SAH in both humans and animal models. However, hippocampal atrophy may not be present in all neuroimaging investigations of experimental SAH. For instance, a recent MRI study by Atangana et al. (2015) did not detect hippocampal atrophy in their experimental model of SAH. This discrepancy between our findings and the results of Atangana et al. (2015) study could be ascribed to the less severe course of tissue injury in their study.

An important question raised in this context could be what might cause hippocampal atrophy following SAH. By considering the key involvement of AQP4 in the maintenance of tissue water homeostasis and ECS volume (Amiry-Moghaddam et al., 2003), it can hence be hypothesized that reduction in AQP4 channels and the consequent changes in the water homeostasis, can entail changes in hippocampal volume. However, in the current study, there was no significant correlation between changes in the length density of AQP4 positive capillaries and the reduction in the volume of the hippocampus. Therefore, it is conceivable that the smaller volume of the hippocampus in this study cannot seemingly be ascribed to the imbalance in the water content of the tissue. In contrast, we observed a positive correlation between the reduced volume of the hippocampus and the diameter of the hippocampal capillaries. Indeed, previous reports have demonstrated SAH-related reductions in oxygen extraction efficacy and cerebral metabolic rate of oxygen (CMRO_2_) (Carpenter et al., 1991; Ohkuma et al., 2000). Earlier observations also indicate that functional and morphological alterations of the cerebral capillaries can result in tissue hypoxia and neurodegeneration after SAH (Ostergaard et al., 2013). Accordingly, we can speculate that reduced diameter of the hippocampal capillaries following SAH can possibly result in hypoxic-ischemic hippocampal injury contributing to the decrease in the hippocampus volume.

In the present study, we observed a positive correlation between changes in the astrocyte morphology including the lower number of astrocyte branches and hippocampal volume loss 4 days following SAH. It has been suggested that conditions which involve glial remodeling are associated with changes in the hippocampus volume (Czeh and Lucassen, 2007), and a recent publication of our group indicated that changes in the volume of the hippocampus are correlated with the morphological alteration of astrocytes (Ardalan et al., 2017). Therefore, we proposed that the observed hippocampal shrinkage in the SAH animals might reflect microstructural changes including astrocytes. Such changes are likely detectable using diffusion MRI techniques sensitive to microstructure such as the clinically feasible fast diffusion kurtosis techniques developed recently (Hansen et al., 2013, 2016, 2017; Hansen and Jespersen, 2016). This work is ongoing in our group.

As a limitation for the current study, brain damage in this animal model can occur as the result of brain tissue being “stabbed” by the filament used to induce the bleeding. Although we attempted to avoid this type of brain injury by the prompt retraction of the monofilament right after the ICP increase, this method-dependent damage cannot be completely avoided. Bleeding severity and the amount of the blood released into the subarachnoid space cannot be properly monitored in this model, since this is mainly dependent on the force and speed applied when perforating the vessel. Thus, SAH severity can differ between animals, leading to variation between experiments.

Another limitation of the current study is the lack of behavioral tests to assess memory deficits and cognitive dysfunctions, indicating secondary brain injuries after SAH. Due to the technical issues, immunolabelling of pericytes was not performed in this study. Considering the potential importance of pericytes in regulating capillary diameter, lack of pericyte morphological investigations should hence be considered as another limitation. Because of the limitation for measuring the total AQP4 protein level of the hippocampus, it remains uncertain how the total AQP4 protein abundance in the hippocampus will be affected after SAH. As SAH induction carried a high mortality, the morphological quantification had to be performed on the limited number of animals. Additionally, applying the stereological methods requires a statistically accurate number of sections from the organ, therefore, limiting our opportunities to perform additional immunostaining for instance CD31, which could provide important information regarding endothelial cells and the degree of angiogenesis. Our study shows that the profound morphological alterations of hippocampal astrocytes correlate with the loss of capillary astrocyte endfeet coverage, as a potential source of reduced hippocampal capillary diameters. The subsequent hippocampal atrophy may provide insights into the etiology of cognitive dysfunctions inflicting SAH survivors, and disturbances at the glia-vasculature interface might account as an important pathophysiological mechanism involved in the development of long-term cognitive sequela after SAH.

## Author Contributions

The main idea of this study was from LØ and BH. MAr, MAn, and NI designed the study and wrote the protocol together. Data collection and data analysis has been done by MAr, MAn, and AR. All authors contributed in writing the manuscript and approved the final manuscript.

## Conflict of Interest Statement

The authors declare that the research was conducted in the absence of any commercial or financial relationships that could be construed as a potential conflict of interest.
